# A scoping review of policies promoting and supporting sustainable food systems in the university setting

**DOI:** 10.1186/s12937-020-00617-w

**Published:** 2020-09-10

**Authors:** Amanda Grech, Eloise Howse, Sinead Boylan

**Affiliations:** 1 School of Life and Environmental Sciences, Faculty of Science, The University of Sydney, Sydney, NSW Australia; 2 Charles Perkins Centre, The University of Sydney, John Hopkins Drive, Camperdown, NSW 2006 Australia; 3 School of Public Health, Faculty of Medicine and Health, The University of Sydney, Sydney, NSW Australia

**Keywords:** Sustainability, Food supply, Food systems, Environmental policy, Institutional policy, Fair-trade, Food waste, university

## Abstract

**Background:**

Transitioning towards sustainable food systems for the health of the population and planet will require governments and institutions to develop effective governance to support the adoption of sustainable food practices. The aim of the paper is to describe current governance within Australian and New Zealand universities designed to support sustainable food systems.

**Methods:**

A systematic search of governance documents to support sustainable food systems within Australian and New Zealand universities was conducted. Data were obtained from 1) targeted websites 2) internet search engines and 3) expert consultations. Inclusion criteria consisted of university governance documents including by-laws, policies, guidelines, frameworks, and procedures that support sustainable food systems.

**Results:**

Twenty-nine governance documents across nineteen Australian and New Zealand universities were included for synthesis, including waste management policies (*n* = 3), fair-trade/procurement policies (*n* = 6), catering and or event guidelines (*n* = 7) and catering policies (*n* = 2), and environmental management plans (*n* = 11). The main strategies adopted by universities were sustainable waste management and prevention (e.g. reducing landfill, reducing wasted food, (27%)), ethical procurement practices (i.e. fair-trade (27%)) and environmentally sustainable food consumption (e.g. local, seasonal, organic, vegetarian food supply (14.5%)). Only 12.5% of universities addressed all three of the main strategies identified.

**Conclusions:**

This study indicates that while sustainable food systems are considered in some university governance documents, efforts are predominantly focused on aspects such as waste management or procurement of fair-trade items which as stand-alone practices are likely to have minimal impact. This review highlights the scope of universities to provide strong leadership in promoting and supporting sustainable food systems through holistic institutional policies and governance mechanisms.

## Introduction

The global food supply has a substantial impact on health of the humans and of the planet; not only do unhealthy diets make a substantial contribution to morbidity and premature mortality, but the current global food production creates the largest environmental burden caused by humans and is threatening ecosystems [1]. Food production not only contributes approximately 30% of greenhouse gas emissions (GHGe), it is also associated with the production of solid and water waste, air emissions and pollutants from farming practices [2, 3]. Opportunities exist to reduce the environmental impact at every step of the food-system including processing, distribution, marketing and consumption practices [3, 4]. By transitioning to healthy and sustainable diets, an estimated 11 million lives can be saved annually in addition to helping avoid severe environmental degradation [5].

The steps that are necessary to ensure the health of the population and the planet have been increasingly articulated, with food and nutrition featuring heavily in the United Nations (UN) sustainable development goals and the Paris Agreement [5]. To help reach these goals, the 2019 EAT-*Lancet* commission set two targets: to transition to healthy diets, outlining goals to double intake of fruits, vegetables, legumes and nuts and halve intake of other foods including sugar and red meat by 2050; and re-orienting global food production to sustainable production, which will require radical improvements to water efficiency, fertilizers, phosphorus recycling, nitrogen and phosphorus redistribution and biodiversity [5].

However, despite the concerted efforts of individuals, large-scale change by organizations and governments is required, particularly at the systems level. Universities are regarded as a specific type of setting that can influence a range of health behaviors including dietary habits, physical activity and tobacco smoking [6, 7]. It is therefore unsurprising that universities have been the focus of attempts to support sustainable food systems [6]. The World Health Organization initiated the Health Promoting Universities framework in recognition that universities are large organizations that have the potential and responsibility of promoting health for staff and students through their organizational culture, structures and practices [8]. In Australia, presently, 8% of the adult population aged 15–64 years (predominantly young adults) are enrolled in university courses (1.3 million people) [9] and 60% of school leavers attend university in New Zealand [10]. Given the size of universities and the vulnerability of young adults to poor diet quality and weight gain [11, 12], universities are uniquely placed to demonstrate leadership in supporting healthy and sustainable food systems.

Universities have historically played a key leadership role in environmental sustainability and since the 1970s many have committed to ‘greening campus’ operations [13, 14]. Traditionally universities’ environmental sustainability efforts have focused on energy, water and resource efficiency and conservation, waste management and recycling [13, 14]. There is limited research on the extent that universities have implemented policies or other governance that supports sustainable food systems. In Canada, reviews of sustainability governance within universities indicate that food is referred to in governance documents, however these reviews did not examine in detail how institutions incorporated this into their governance [15, 16]. Campus food sustainability projects in the USA have reportedly increased in recent years spurred by campus audits that show food procurement and transportation contribute substantially to GHGe [17, 18]. In North America, the ‘Sustainability Tracking, Assessment & Rating System (STARS)’ is a self-reporting framework for colleges and universities to measure their sustainability performance. Several institutions have signed up to the STARS in Canada and the USA, while many institutions have publicly available food sustainability policies in the UK [18, 19]. In Australia, student-led groups such as the Fair Food Challenge are working to create healthy, sustainable and fair campus food systems [20]. A search of the grey literature has shown that universities in New Zealand and Australia have publicly accessible sustainability governance, however to our knowledge, there is no evidence of specific food sustainability policies currently implemented in the region. As both countries have some of the highest GHGe emissions per-capita in the world, they are key regions to focus efforts on sustainable food-systems [21].

This study aims to describe current governance strategies and policies within Australian and New Zealand universities that aims support to sustainable food systems.

## Methods

A systematic search of grey literature for food sustainability governance documents within universities in Australia and New Zealand was conducted. The protocol for this review including the research question, inclusion and exclusion criteria, data sources and search strategy, and the process for selection of the evidence, data-charting and synthesis was determined a priori. This was done in accordance with the PRISMA Extension for Scoping Reviews (PRISMA-ScR) checklist, peer-reviewed journal articles on applying systematic search methods to grey literature were used to inform the search as described below [22–26].

### Inclusion/exclusion criteria

Governance documents were defined as: *“a formal statement of intent that mandate principles or standards that apply to the University’s governance or operations or to the practice and conduct of its staff and students”* [27]. The FAO definition for a sustainable food system was used: *‘a food system that delivers food security and nutrition for all in such a way that the economic, social and environmental bases to generate food security and nutrition for future generations are not compromised*’ [28]. Inclusion criteria consisted of 1) governance documents in which laws, by-laws, policies, guidelines, frameworks, and procedures that relate to the sustainable food systems were described and 2) universities located within Australian and New Zealand. Superseded or rescinded policies were excluded from this review.

### Information sources

As recommended in the literature, the information sources to identify relevant governance included: 1) Targeted websites - a list of all of the universities with-in Australia and New-Zealand were obtained from government websites to ensure that all universities were included. Each university website, including the university’s policy/governance libraries, were hand-searched for relevant policies; 2) Internet search engine (i.e. customized Google searches). The first 100 search results were screened for inclusion, as suggested by others [23, 25]; 3) Expert consultation - targeted emails were sent to university policy officers and/or relevant academic staff.

### Search

Search terms and synonyms included (food or catering or nutrition or diet) AND (sustainable or environment or procurement or fair-trade) AND (policy or guideline or procedure or framework or governance) AND (university or college or campus). For the general Google search, the search results were restricted to content from Australia and New Zealand and the search was repeated for each country separately.

### Selection of evidence

Documents were initially screened for relevance by title, executive summaries, or table of contents. Full-text documents were then read to ensure the documents met the inclusion criteria. Duplicate documents were removed. Two reviewers screened a sample of eligible studies and achieved good agreement (90%), and one reviewer screened the remaining articles.

### Data charting and synthesis

As per recommendations for systematic searches of the grey literature [23], an excel spreadsheet was constructed which listed the name of each university and the URL to the relevant web pages. Data were extracted from each of the policies into a standardized form, which was piloted to ensure that relevant data were extracted. Information collected was: Year of publication, document type, topic, document purpose, details on how compliance was assessed, details of any working groups to oversee the execution of the governance aims, the major theme of the document, and specific sustainability strategies outlined. The included governance documents were synthesized in a narrative review and results were crosschecked by two researchers.

## Results

**Fig. 1:**
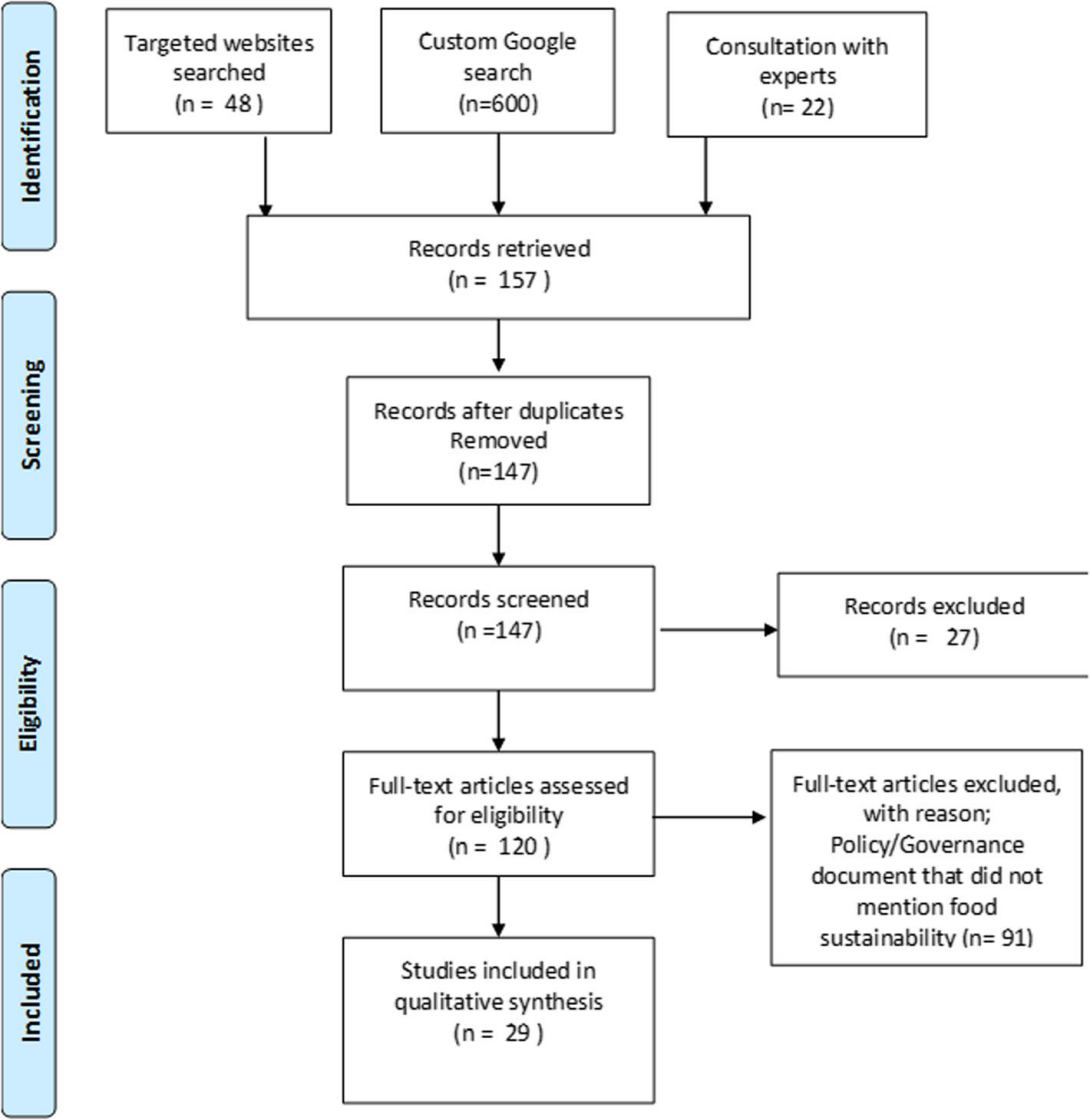
Flow diagram of the search process

A total of 48 Australian (*n* = 40) and New Zealand (*n* = 8) university websites were searched. Nineteen universities (16 from Australia, 3 from New Zealand) with governance documents relating to sustainable food systems were found. From these universities, there were 157 documents located in the initial screening process and 29 documents were included for synthesis As per the recommendations for conducting scoping reviews of grey literature, an internet search engine was used in conjunction with staff consultation; this search only produced a small number of results that duplicated the targeted website search or otherwise did not meet the inclusion criteria. Other reasons for exclusion were: having a sustainability policy that did not refer to food; governance documents that were not from the broader university setting (e.g. produced by the university childcare center); and other documents that relate to food sustainability but were not governance documents (e.g. promotional materials). For a flow-diagram of the search process, see Fig. 1.

The governance documents included catering policies (*n* = 2), fair-trade policies (*n* = 4), procurement policies (n = 2), catering and/or event management guidelines (*n* = 7), waste management plans (*n* = 3), environmental management plans (n = 2), environmental procedures (*n* = 1) and sustainability strategic frameworks (*n* = 8). Here we will describe the types of governance used (Table 1), followed by the key target areas and specific strategies outlined in these documents (Table 2).

**Table 1:** Governance documents – aims, implementation and target areas of Governance in Australian and New Zealand universities relating to sustainable food systems

Years	Document Type	Topic	Document Aims	Working Groups	Compliance	Target Areas^a^
2015	Policy	Catering – Managerial Policy	Defines the purposes and conditions of catering	Chief Operating Officer	Chief over-seeing officer responsible for approving all catering orders over $80	WM
2016	Policy	Sustainable (catering) procurement standards	Sustainable procurement considers the broader economic, environmental, and social cost of purchases made by the university	Nil mentioned	Procurement records will be scrutinized internal and external audits	WM, EP, SFC
2012	Policy	Fair-Trade	Describe conditions of Fair-trade on University	Fair-trade Steering Committee	Fair-trade accreditation	EP
2010	Policy	Fair-trade	To outline activities to maintain fair-trade accreditation	Fair-trade steering committee	Nil mentioned	EP
2014, 2017	Policy	Fair-trade	To outline how Fair-trade will be embed into the university	Fair-trade Action Group,	Disciplinary action may be actioned for breaches. Fair-trade accreditation	EP
2011	Policy	Fair-trade	Outline fair-trade procedures	Sustainability staff; Student engagement team	Fair-trade university accreditation.	EP
2016	Policy	Procurement	University policy for acquiring goods, services and works	Fair-trade Steering Group	Fair-trade accredited	EP
2012	Policy	Procurement	Ensure products purchased with university funds are procured sustainably	Sustainability committee	Fair-trade accreditation	EP
2017	Guidelines	Healthy catering	Healthy catering guidelines for catered university events	Healthy eating’ working group	Compliance self-assessment checklist	SFC
2017	Guidelines	Sustainable Catering	Outline sustainable catering practices	Sustainability working group	Staff worked with caterers to achieve goals	WM, EP, SFC
2009	Guidelines	Sustainable Catering	To ensure that catering is sustainable.	Sustainability coordinators	Voluntary	WM, EP, SFC
2017	Guidelines	Sustainable Events	Outline sustainable catering practices	Sustainability working group	Nil mentioned	WM, EP, SFC
2014	Guidelines	Sustainable Events	Outline sustainable event practices	Sustainability coordinators	Voluntary	WM, EP, SFC
2017	Guidelines	Sustainable events	Outline sustainable catering practices	Fair-trade Steering Committee, Sustainability committee	Sustainability self-assessment tool	WM, EP, SFC
2013	Guidelines	Sustainable Events/Meetings	To conduct events in line with the universities strategic plan	Nil mentioned	Nil mentioned	WM, EP, SFC
2014	Plan	Environmental Management	Describe the actions taken in the environmental management plan and the carry forward strategies to improve Energy & Greenhouse gases, water usage & disposal, indoor environment, transport and waste and recycling	University Sustainability Committee	All strategies are assessed for completion and reported to the sustainability team	WM
2016–2018	Plan	Environmental Management	Describes the strategy for improving sustainability including Energy and greenhouse gas emissions management; Water conservation; Waste management; Compliance and pollution prevention; Campus environment, biodiversity, and open space; Integration, communication, and engagement; Transportation	Waste Facilities Manager; UNSW Sustainability management	Annual Sustainability Report	WM
2014	Plan	Waste Management	Describe actions required to reach goal of zero waste on campus	Carbon Compliance Reporting and Performance Group; Sustainability staff and student engagement teams	Quarterly waste audits/ Carbon reporting	WM
2015	Plan	Environmental Management	Outlines the actions required for environmental management in relation to campus operations	Environmental Supervisor, Manager of Cleaning Waste and Recycling	Audit of waste produced, landfill reduced and recycling amount	WM
2015	Plan	Waste Management	Reduce waste generation through the five-hierarchy system of waste: prevention, minimization, recycling, energy recovery, disposal	Estate director	Waste auditing required in all waste and recycling contracts	WM
2014	Procedures	Sustainability	To link documents related to strategies for university wide sustainability (i.e. fair-trade policy and catering guidelines)	NA	NA	WM, EP
2017	Strategic Framework	Sustainability	Describes the ways the university will embed sustainability into the university – whole systems approach including operations, research, teaching, community, and engagement and acting as a catalyst for change	Working party on environmental sustainability	Not mentioned	WM, EP, SPC
2017	Strategic Framework	Campus Sustainability	To describe the sustainability programs/imitative and goals relating to carbon reduction, water efficiency, training and skills, engagement activities, sustainable procurement, and investment	University Sustainability Board	Sustainability reporting, Policies state mandatory and voluntary reporting goals	WM, EP
2010, 2017	Strategic Framework	Climate	Inventory of greenhouse gas emissions of the university and action plan to reduce emissions by 20% in 2016 and 100% by 2038. Relevant to Food; reduce solid waste	Sustainability Coordinator and Carbon Monitoring Task Force	Campus Carbon Calculator, annual reports	WM
2017–2021	Strategic Framework	Environmental Sustainability	To embed sustainability with the university through teaching learning, research, governance, operations, and engagement. Lead by example by supporting sustainable food systems	Sustainability executive membership, Fair-trade steering committee	Annual voluntary reporting	WM, EP
2012	Strategic Framework	Sustainability	Outline the universities sustainable goals in keeping with the strategic plan.	The Vice Chancellors Sustainability workforce	Waste audits	WM, EP
2012	Strategic Framework	Sustainability	To embed sustainability into the university’s activities: teaching, research, bridging programs to encourage high-school students to study sustainability, collaborate with the community, staff development, branding, reducing carbon emissions, sustainable campuses -water, grounds & food, waste & procurement and purchasing	Sustainability Working Group	Not mentioned	EP, SFC
2017	Strategic Framework	Sustainability	To outline how the university will embed sustainability into the plans, policies, processes, and procedures 2017–2020	Sustainability sub-committee, director of campus life	KPI’s incorporated group and faculty level. Reviewed annually	WM, EP
2014	Strategic Framework	Sustainability	Sustainability strategy to outline how the strategic plans will be carried out	Sustainability working group; The reporting Action Group	Sustainability will be incorporated into KPI’s	WM, EP, SFC

^a^Target areas include: *WM* Waste Management, *EP* Ethical Procurement, *SFC* Sustainable food consumption

**Table 2:** Strategies outlined in Governance documents to support sustainable food systems

**Strategies (n = universities)**	
***FOOD WASTE MANAGEMENT AND REDUCTION (n = 13)***	
***Prevent food waste (n = 9)***	
Serve appropriate portion sizes to minimize waste	
Give leftovers to participants to take home or share within the university or donate to charity	
Store foods appropriately to maximize shelf life	
At events, cater for correct numbers (i.e. RSVPs rather than estimates)	
Implement strategies to reduce food waste	
Work with retailers to reduce food waste	
Engage food waste charities in community events on campus to increase student engagement in reducing food waste	
Accommodate dietary requirements to avoid waste	
***Dispose of food waste in a sustainable manner (n = 13)***	
Food waste composting to increase by a total 30%	
Cooking oil used on campus must be processed into biodiesel	
Compost/use worm farms to dispose of food waste	
Reduce food scraps in land-fill composting and collecting scraps for pig food	
Food waste should be composted if rescue was not possible	
***Minimize packaging (n = 5)***	
Reduce bottled water by providing water filling stations and discouraging staff and students from single use plastic water bottles	
The purchase, sale and distribution of single use plastic water bottles are not permitted in any university facility.	
Offer an incentive/promote re-usable cups	
Serve tap water not bottled water	
Use reusable crockery	
Choose foods with minimal packaging	
Flavored beverages (except where no alternative exists) must be in non-plastic materials	
Serve sugar, salt, and condiments in reusable dishes, rather than packet	
***Recycle packaging (n = 13)***	
Outlets and catering services must use reusable catering supplies and ensure compatible with the university recycling system	
Increase practical options for recycling	
Improve recycling facilities in dinging areas	
Develop a policy for campus suppliers to reduce packaging and cease use of polystyrene	
Work towards caterers using only recyclable food packaging that can be recycled onsite	
Develop and monitor practices that will reduce waste to landfill and increase recycling	
Use biodegradable and/or recyclable packaging products	
***General (n = 1)***	
Implement a comprehensive behavior change campaign to ensure staff and students use the systems in place to reduce waste	
**ETHICAL PROCUREMENT (*n*** **= 12)**	
***Steering committees (n = 5)***	
Establish a fair-trade steering committee	
***Fair-Trade canteen consumables (n = 12)***	
Select fair-trade tea, coffee, and hot chocolate (when available)	
Make (only) fair-trade products (tea, hot chocolate, coffee, chocolate, and nuts) available across all campus outlets/at staff events	
Encourage the voluntary establishment of the ‘fair-trade kitchenette’. A representative staff member overseas and monitors staff response to transiting to fair-trade products in the office	
Only work with companies that already support Fair-Trade	
Embed Fair-Trade into catering policies	
Work with campus suppliers to ensure Fair-trade is provided at all university events	
University preferred suppliers will only provide Fair-trade canteen consumables	
Make (only) Fair-trade Certified Products available at meetings and in office kitchens	
***Community Engagement (n = 8)***	
Regularly display promotional materials for fair-trade offerings (cafes/events/in newsletters/noticeboards)	
Increase student engagement and support in fair-trade	
Develop engagement strategy for increasing discussion of fair-trade issues within academia and bring together all relevant interested parties	
Celebrate fair-trade fortnight on Campus	
***Fair-Trade accreditation (n = 4)***	
Achieve and/or sustain fair-trade status	
Create a sustainable procurement policy that includes food (i.e. fair-trade)	
Support fair-trade	
**ENVIRONMENTALLY SUSTAINABLE FOOD CONSUMPTION (n = 8)**	
***Seasonal, local, organic food (n = 5)***	
Use seasonal produce from local suppliers (list of fruit and vegetables provided for caterers)	
Expand edible landscaping and promote the benefits of local food	
Provide food grown on campus to food insecure students or to supply campus cafes	
Increase the availability of locally sourced and sustainably produced foods on campus	
Supply food produced by environmentally friendly production methods i.e. organic certified food	
On-campus caterers use local and seasonal produce	
***Animal products (n = 5)***	
Provide vegetarian options	
Minimize the use of meat, dairy and eggs	
***Sustainable seafood (n = 4)***	
Use sustainable seafood (i.e. sustainable fish stocks caught with methods that do not harm the environment)	
***Health and well-being (n = 3)***	
Provide foods that are healthy and promote well-being	
***General (n = 5)***	
Use the university approved sustainable catering services that adhere to the principals of sustainable catering	
Introduce beehives as part of a biodiversity management plan	
Support sustainable procurement including catering	
Engage with all sectors of the community that drive sustainability e.g. provide grants for community gardens, hold a farmer’s market on campus for local seasonal food	
Enhance efforts to interest the university community in sustainably produced foods	
Use campus engagement initiatives e.g. support for a student led initiative to create a sustainable food system and calculate nitrogen release from food production	
Promote sustainable initiatives at events/to consumers	
Participate in research and include curriculum to address risks including climate change, energy demand, water scarcity, population growth, food security and ecosystem decline	
Work towards integrating sustainability criteria into contracts including dining services	
***ENERGY AND WATER CONSERVATION (n = 2)***	
Caterers on campus agree to meet water conservation and energy efficiency standards	
Use fresh, not frozen vegetables to reduce energy costs	
Use energy efficient appliances for catering	
Conserve water use during catering and clean-up (install water saving device, water efficient dishwashers) and benchmark progress	

### Governance and compliance

Several different types of governance were identified with each having different levels of enforcement (Table 1). These were policies, guidelines, plans and strategic frameworks. Policies state a course or principle of action adopted by the universities concerning food sustainability goals. They mandate rules for the university’s operations and were assessed for compliance (7 out of 8) with audits (*n* = 2), accreditation boards external to the university (*n* = 4) or mentioned the use of disciplinary action for breeches (*n* = 1). Guidelines provided suggestions or instructions on how to achieve more environmentally sustainable catering and events on campus and could be voluntarily adopted (*n* = 7). Waste management and environmental management plans provided an inventory of the strategies to minimize the environmental impact of campus operations. All plans provided metrics to measure the success of the strategies implemented and were measured with audits or annual reports. Strategic frameworks contained institutional statements on the commitment to environmental sustainability and the overarching goals of the university and how they would achieve these goals. Six of the strategic frameworks provided details on how achievement of the goals would be assessed including voluntary and mandatory reporting or audits (*n* = 4) and two mentioned the use of key performance indicators (KPIs).

### Key target areas

Three key target areas related to sustainable food systems were identified in the governance documents including food waste management, fair-trade and environmentally sustainable food consumption (Table 1). Food waste management and reduction were the most common theme of sustainable food systems governance with 13 universities considering this in 22 documents. Twelve universities published 21 governance documents related to ethical procurement, which detailed the use and promotion of fair-trade products. Eight universities published 11 governance documents that adopted strategies directly related to environmentally sustainable food consumption e.g. the use of animal products or local produce. Only 12.5% of universities addressed all three of the target areas. Two universities also briefly addressed water conservation and energy efficiency of food service on campus.

### Strategies outlined in the governance documents

#### Food and food packaging waste management and reduction

Waste management strategies covered two main areas, including those that aimed to prevent and reduce food wastage from occurring and those that aimed to improve the management associated food waste products (e.g. food packaging, coffee cups and food scraps). The most common food waste reduction strategies were confirming attendance numbers for events; donating and/or sharing leftovers with event attendees; and student engagement activities. Eleven universities implemented strategies that aimed to increase the amount of food scraps and/or food and beverage packaging that would be diverted from land-waste. Examples include composting or use of minimal/recyclable food packaging only, particularly coffee cups or single-use plastics. Waste management strategies are listed in Table 2.

#### Ethical procurement

The most common aim of fair-trade governance was to increase the use of fair-trade canteen consumables, particularly tea and coffee, used on campus in staff rooms, for events and in outlets (Table 2). To achieve this, it was commonly stated that the availability of fair-trade would be increased to minimum thresholds in campus outlets as well as adopting student and staff engagement strategies to increase the use of fair-trade products. Forming fair-trade committees to oversee the university’s fair-trade activities were frequently mentioned.

#### Environmentally sustainable food consumption

Universities adopted several strategies that directly related to improving the environmental sustainability of catering or food available on campus (Table 2). The use of seasonal and/or local produce was the most frequently encouraged strategy including the use of community gardens on campus, encouraging event organizers to source local food for staff catering and ensuring on campus food outlets only used local food. Other strategies included the use of foods produced with environmentally friendly production methods (e.g. organic certified and sustainably sourced seafood) and minimizing the use of animal products available at events catered with university funds and/or available for sale on-campus (Table 2). Community engagement or awareness-raising initiatives were also adopted by some universities including providing grants for community gardens, encouraging event organizers to promote sustainable initiatives adopted at events and supporting student-led initiatives that aim to support sustainable food systems.

## Discussion

Approximately one-third of universities in Australia and New Zealand addressed at least one component of sustainable food systems within their university governance. All the universities’ governance documents considered sustainable food systems primarily through campus operations rather than other activities under a university’s remit, such as education and research. Most university governance considered only waste management (i.e. reducing landfill or reducing food waste) and/or the use of fair-trade products. These were predominantly outlined within university strategic frameworks, campus management plans and policies and described how the policy would be enforced or assessed for compliance with metrics such as KPIs or audits. Less emphasis has been given to supporting a shift towards dietary patterns (e.g. reducing consumption of red meat and substituting it for healthier plant-based foods), with only 15% of Australian and New Zealand universities currently employing such strategies. These practices were mainly included in guidelines rather than policy documents and were generally a consideration or implemented voluntarily. This review demonstrates the scope for universities to have a positive impact in the transition towards a sustainable food supply, however, it also highlights the need for governance that works towards national and international goals for healthy and sustainable food systems.

Many university policies addressed single issues, such as reducing single-use plastics, composting food scraps or using fair-trade canteen consumables and only 12.5% of universities have adopted strategies from all three key target areas identified in this review. While each of these strategies is important, they do not single-handedly support sustainable food systems and more holistic approaches are needed. Lawrence et al. (2019) argues that ‘nudges, adjustments or tweaks’ are insufficient on their own to achieve healthy, sustainable diets and food systems [29]. This problem is not isolated to food sustainability policy specifically but has plagued sustainability policy within higher education institutions, which have traditionally focused on specific aspects of sustainability (e.g. water usage, air emissions and waste management) without considering the complexity of achieving environmental sustainability [13]. A systematic process that considers the multi-dimensional nature of environmental sustainability is required but rarely implemented [13]. Systems thinking that applies a “whole of university approach” to sustainability and health has been recommended to maximize universities’ capacity as agents of change [6, 13, 30]. This involves adopting sustainability goals that include the three primary domains including: sustainable university operations; sustainability curriculum and research; and participation by the university community and community outreach [6, 13, 30]. A commitment to food sustainability curriculum and research is needed but largely absent from university governance in Australia and New Zealand.

The most common goal was to reduce the environmental burden of food packaging. This mirrors research findings on Australian consumer perception on the most important interventions needed to achieve an environmentally sustainable food supply [31]. Ninety percent of consumers ‘strongly agreed’ or ‘agreed’ that food manufacturers reduced food packaging is beneficial for improving the environment, while only 22% of consumers agreed that reducing meat consumption was important [31]. This suggests many consumers are unaware of other important issues surrounding food sustainability and food systems and there has been slow uptake of sustainable practices within the community [31–33]. It could explain why reducing packaging is frequently a goal of university sustainability governance documents. The lack of awareness and understanding from consumers has been named as a key barrier in initiating a healthy, sustainable and safe food policy in Australia by Australian policymakers [34]. Knowledge is a necessary (although not sufficient) component for behavior change and consumers must understand why behavior change is necessary before they can be motivated to make the required changes [30]. Consumers also require supportive environments to help with shifting choices and behaviors regarding food [35]. This research suggests that greater education of the public regarding environmental impacts of the food supply is a critical first step for creating more sustainable food systems.

The FAO has adopted the definition of sustainable diets that includes three pillars of sustainable food systems: economic sustainability, environmental sustainability, and social and cultural sustainability [28]. The findings from this current study indicate that universities have not holistically considered sustainability, with most of the strategies only focusing on environmental protection. This is not surprising given recent research has indicated that policy actors in Australia define sustainability within a narrow scope [36]. Some suggest that in practice it has proven difficult to assimilate the more holistic definition of sustainability due to disparate methodologies of the disciplines involved [37].

### Food waste reduction and management

#### Reducing food waste

Reducing food wasted in the university setting was primarily attempted for catered events rather than for food outlets. Examples of strategies included simple measures to reduce waste, such as ensuring attendance was by RSVP for accurate catering estimates, distributing leftovers to guests and donating food to charity. Food rescue charities have become increasingly popular and are now partnering with universities in improving community engagement and redistributing good quality food [38]. Although it is not certain what proportion of food is rescued in universities, an estimated 18,105 tons of food is rescued annually in Australia [39]. Plate waste is also an important component of wasted food, and USA initiatives that have removed trays from cafeterias have seen a 20% food waste reduction [40]. One governance document described employing this strategy but found it to be ineffective, attributing the lack of success to differences in foodservice and lack of buffet-style dining. Research suggests that ‘nudging’ practices to reduce food waste, such as serving smaller portions, can successfully reduce plate waste and food intake [41].

#### Food waste and food packaging disposal

University campuses used a variety of waste management programs for food waste unfit for human consumption. These included converting scraps into fertilizer, recycling food scraps through composting or using it for animal feed, demonstrating that such efforts are feasible on a university campus. As a model for informing food waste management, the Australian Government’s ‘National food waste strategy’ has partnered with an Australian university and structured the food waste policy around a hierarchical model of preferential treatment of food waste management. This model places the greatest priority on preventing waste from occurring, as this will have the greatest beneficial impact on the environment [42]. However, if waste occurs, sending food and packaging to landfills should be a last resort as it’s a major contributor to GHGe [42]. Alternatives to disposing of waste in landfills include recycling, recovering resources (e.g. converting food to fertilizer or digested anaerobically and injected into the gas grid) or degrading the food by anaerobic digestion through composting or worm farms.

#### Ethically sourced food and beverages

Around a quarter of Australian and New Zealand universities adopted fair-trade governance including stand-alone fair-trade policies. The primary aim of fair-trade products is to ensure that workers and farmers receive a fair price for their products and good working conditions [43, 44]. The rationale for including the use of fair-trade canteen consumables in environmental sustainability governance is that fair-trade products must also meet environmental protection and climate change standards [44, 45]. These standards require adopting farming practices that aim to minimize energy and greenhouse gases, protect the quality of water and soil, prohibit the use of particular harmful chemicals and genetically modified organisms, adopt best practice waste management and aim to protect biodiversity [43]. The Fair-Trade Association of Australia and New Zealand accredits universities as a ‘Fair-Trade University’ and perhaps explains why there was greater uptake of governance has been surrounding fair-trade compared to other possible strategies. However as fair-trade certification and therefore policies only apply to canteen consumables (such as tea, coffee, and chocolate), there is room for fair-trade policies to consider the broader food system.

#### Sustainable food consumption

Methods to create a sustainable food supply on campus were reducing animal products; increasing the provision of vegetarian and vegan options; and preferencing the use of local, seasonal, and organic produce.

In Australia and New Zealand, ruminant meat makes a considerable contribution to agricultural GHGe [4]. There would be health advantages to reducing red meat which is currently 24% (565 g of red meat per week) over the Australian Dietary Guidelines recommended intake to prevent bowel cancer [23]. Red meat and processed meat make a significant contribution to colorectal cancer incidence in New Zealand [29]. Two significant challenges to effective policies that aim to reduce meat intake, is the lack of consumer awareness regarding the benefits of replacing red meat with alternatives and the lack of willingness to reduce meat internationally including Australia, Germany, Netherlands, Portugal and the USA [7, 30]. One university governance document described the implementation of ‘Meatless Monday’ in the cafeterias and providing vegetarian options, however due to resistance from staff and students, the policy was retracted, reflecting the degree to which consumers may be resistant to reducing meat consumption. Strategies for reducing meat consumption may need to be less restrictive to increase acceptability [30]. Experimental evidence suggests that nudging interventions that gently encourage consumers to reduce meat may be effective [46]. These include reducing the portion size of meat offered, providing meat alternatives along with educational materials, and changing the sensory properties of meat alternatives that may prove effective at managing demand [46].

Regarding organic produce, research which takes a food systems is needed to more accurately quantify sustainability outcomes [47]. It has been suggested that a combination of food sub-systems is required to achieve the most sustainable food system. For example, industrially produced chicken leads to lower CO2 emissions than civically (i.e. household or community) produced chicken [47]. This is contrary to the assumption that local food is always more sustainable and highlights the need for greater research on sustainable food systems to inform evidence-based policy [47].

#### Implementation and evaluation of governance

It is currently unclear whether the governance strategies identified in this study did indeed help achieve more healthy and sustainable food systems within university settings. Policies have been criticized in the past for “having no teeth”, as policies are statements of intention that may not translate into actions [31, 32]. It is recommended that strategies are evaluated to determine whether policies are being implemented as intended (process evaluation) and whether such policies are having an impact on health and environmental outcomes (impact and outcome evaluations). This is particularly important for policies that may have unintended consequences or misalignments between health and sustainability. For example, banning bottled water without the provision of alternative ways to access water may encourage consumption of sugar-sweetened beverages, which is undesirable given the relationship between sugar-sweetened beverage consumption and obesity [48].

##### Limitations

While every effort was employed to ensure a comprehensive assessment of university governance using robust search strategies, it is possible that some documents were not located during the search stage, particularly if they were not publicly available documents. It is important to note that the paper aimed to describe the content of governance documents and therefore does not attempt to capture all university initiatives to create sustainable food systems. Some initiatives and practices such as community gardens may not be captured if they were not outlined within university governance documents. Furthermore, only 19 out of 48 universities mentioned food within sustainability governance documents, however almost all of the universities had environmental sustainability governance; these policies could potentially be applied to contribute to a more sustainable food supply (e.g. sustainable procurement policies).

## Conclusions

This review is the first to document food sustainability governance policies and practices within Australian and New Zealand universities. It highlights the urgent need for a more comprehensive range of strategies to address healthy and sustainable food systems within university settings. Many of the universities targeted a single aspect of a sustainable food system such as the use of fair-trade products or waste management. While important strategies, these standalone practices are likely to have minimal impact on reducing the negative environmental or health impacts of current food systems. In addition, strategies were incorporated primarily into campus operations, however there is scope for better integration of strategies within other university activities, including teaching and research. The findings highlight the need for a more holistic approach to achieving healthy and sustainable food systems within the university setting.

## Data Availability

The datasets used and/or analyzed during the current study are available from the corresponding author on reasonable request.
